# Investigating the Causal Link between Rheumatoid Arthritis and Atrial Fibrillation in East Asian Populations: A Mendelian Randomization Approach

**DOI:** 10.1155/2024/3274074

**Published:** 2024-07-15

**Authors:** Weijun Luo, Hui Yv, Xiao Yu, Xianjun Wu

**Affiliations:** ^1^ Department of Cardiology Lishui People's Hospital The Sixth Affiliated Hospital of Wenzhou Medical University, Lishui, Zhejiang, China; ^2^ Department of Cardiology First Affiliated Hospital of Lishui University School of Medicine, Lishui, Zhejiang, China

## Abstract

**Background:**

Rheumatoid arthritis (RA) has been associated with atrial fibrillation (AF) in observational studies, yet the causal relationship remains elusive. In this study, we employed Mendelian randomization (MR) to investigate the impact of RA on AF risk specifically in East Asian populations.

**Methods:**

Utilizing genome-wide association study (GWAS) data on RA (*n* = 212,453) and AF (*n* = 36,792), we applied the following five MR methods: inverse variance weighted (IVW), MR-RAPS, maximum likelihood, weighted median (WM), and Bayesian weighted Mendelian randomization (BWMR). We evaluated heterogeneity, sensitivity, and pleiotropy.

**Results:**

Five genetic instrumental variants for RA were identified. All MR methods consistently indicated a causal association between RA and AF (IVW: OR = 1.20, 95% CI: 1.01–1.41, *p* < 0.03; MR-RAPS: OR = 1.21, 95% CI: 1.03–1.42, *p* < 0.02; maximum likelihood: OR = 1.20, 95% CI: 1.04–1.39, *p* < 0.01; WM: OR = 1.25, 95% CI: 1.03–1.52, *p* < 0.03; and BWMR: OR = 1.20, 95% CI: 1.02–1.42, *p* < 0.03). Sensitivity and pleiotropy analyses confirmed the robustness and validity of the results.

**Conclusions:**

This study establishes a causal link between RA and AF in East Asians. Our results underscore the need for in-depth mechanistic investigations to unravel the underlying pathways. Clinicians should consider AF risk in RA management, emphasizing collaborative care between rheumatologists and cardiologists. Moving forward, future research should explore therapeutic interventions and address the shared biological mechanisms.

## 1. Introduction

Rheumatoid arthritis (RA) is a chronic systemic autoimmune disorder characterized by inflammation that can affect various parts of the body, including joints, skin, lungs, kidneys, heart, and blood vessels [1, 2]. With a global prevalence ranging from 0.5% to 1.0% and showing no signs of decline, RA stands as one of the most prevalent autoimmune diseases [3]. Extensive research underscores the substantial burden that RA imposes on both individuals and society [4].

Inflammation-related cardiovascular diseases (CVDs) exacerbate the economic burden and mortality rates among RA patients [5]. Notably, atrial fibrillation (AF) manifests significantly more frequently in RA patients compared to healthy individuals [6], indicating a potential link between chronic inflammation and destabilization of myocardial electrophysiology [7, 8]. We hypothesize that a causal relationship exists between RA and AF though their association may also be influenced by shared risk factors, posing challenges for elucidating causality.

To circumvent the biases inherent in observational studies, Mendelian randomization (MR) analysis leverages genetic variants as instrumental variables (IVs) to infer causal relationships [9]. By exploiting the random assortment of genetic variants during gametogenesis, MR simulates randomized controlled trials, enabling investigations into causal questions that are otherwise challenging due to cost or ethical considerations. Given the burgeoning popularity of genome-wide association studies (GWASs) in the past decade, researchers increasingly turn to MR to identify disease risk factors and biomarkers.

Recent studies have also explored the probability of spontaneous conversion to sinus rhythm in patients with symptomatic AF. For instance, a study developed and validated a score to determine the probability of spontaneous conversion to sinus rhythm in patients presenting to the emergency department with hemodynamically stable, symptomatic AF [10]. Another study reviewed the incidence and determinants of spontaneous cardioversion of early onset symptomatic AF, identifying key predictors such as the absence of heart failure, small atrial size, and recent-onset AF [11]. These studies highlight the importance of understanding the factors influencing AF management and outcomes.

In this study, we adopt a two-sample MR design to explore the causal relationship between RA and the risk of AF using large-scale GWAS datasets of individuals with East Asian ancestry. This approach allows us to rigorously investigate the potential causal link between RA and AF while minimizing biases inherent in observational studies.

## 2. Materials and Methods

### 2.1. Data Source

The exposure and outcome datasets were obtained from the Medical Research Council Integrative Epidemiology Unit (MRC-IEU) open GWAS database (https://gwas.mrcieu.ac.uk). A large-scale GWAS meta-analysis involving 212,453 subjects of East Asia ancestry yielded summary statistics for RA (4,199 RA cases and 208,254 controls) (https://gwas.mrcieu.ac.uk/datasets/bbj-a-151/). The outcome variable data for AF were derived from a GWAS meta-analysis including 36,792 subjects of East Asia ancestry (8,180 AF cases and 28,612 controls) (https://gwas.mrcieu.ac.uk/datasets/bbj-a-71/).

The quality of the datasets was ensured through rigorous quality control measures applied during the GWAS meta-analyses. These measures included the removal of individuals with ambiguous sex, high genotype missingness, and excessive heterozygosity. In addition, SNPs with low call rates, deviations from Hardy–Weinberg equilibrium, and low minor allele frequencies were excluded. The datasets were imputed using reference panels from the 1000 Genomes Project to ensure high coverage and accuracy of the genetic variants.

Population stratification has the potential to introduce bias into MR analysis. Because allele frequencies differ, a single nucleotide polymorphism (SNP) may be related to ancestry while also being related to disease risk. To mitigate this bias, SNPs and their corresponding summary statistics in the MR analysis were restricted to East Asia descent for the exposures and outcomes. The details of the data source are listed in Table 1.

### 2.2. Genetic Instrumental Variables (IVs) Selection

The two-sample MR approach relies on the following three key assumptions [12]: (1) the genetic variants must be strongly associated with the exposure; (2) the variants must affect the outcome solely through their effect on exposure; and (3) the variants must be independent of any confounders of the exposure-outcome association. We selected SNPs associated with the exposure (RA) at the genome-wide significance level (*p* < 5 × 10^−8^). Using the PLINK clumping algorithm, we identified independent IVs by calculating the linkage disequilibrium (LD) (*r*^2^ < 0.001) within a physical window of 10,000 kb [13]. Palindromic SNPs with intermediate allele frequencies were removed from both the SNPs-exposure and SNPs-outcome datasets, resulting in a single dataset [14]. To account for potential confounding factors, we searched the PhenoScanner website (http://www.phenoscanner.medschl.cam.ac.uk) to assess previously documented associations with AF or its recognized risk factors [15]. In this study, coronary artery disease (CAD), hypertension, and body mass index (BMI) were identified as confounders when AF was considered as the outcome. We calculated the F-statistic for each IV-SNP using the following equation to avoid weak instrumental variable bias: *F*=*R*^2^(*N*–*K* − 1)/(1 − *R*^2^), where (*R*^2^) represents the genetic variance explained by each SNP, (*N*) is the sample size, and (*K*) denotes the number of SNPs. An F-statistic greater than 10 indicates a significant association between the selected IVs and RA [16].

### 2.3. Mendelian Randomization Analysis

To estimate the causal effect, we performed an inverse variance weighted (IVW) meta-analysis on each Wald ratio. The IVW approach is considered reliable when there is no evidence of directional pleiotropy (*P* for MR-Egger intercept >0.05) among the chosen IVs [17].

### 2.4. Sensitivity Analysis

To ensure the robustness of our causal findings, we conducted a comprehensive sensitivity analysis. First, we rigorously assessed the following four MR methods: IVW, MR-RAPS, maximum likelihood technique, and weighted median (WM). MR-RAPS accommodates even very weak instruments, enhancing our ability to detect causal effects [18]. The maximum likelihood technique assumes a linear relationship between exposure and outcome, directly maximizing the likelihood of estimating causal influence based on SNP effects [16]. Validity of at least 50% of the instruments was ensured using the weighted median approach [19]. Consistency across these methods provides robust evidence for causality, accounting for their distinct assumptions. Second, we examined the impact of individual SNPs on outcomes. In addition, we evaluated heterogeneity using the Cochran's *Q* statistic and the *I*^2^ statistic. Heterogeneity is indicated by a *Q* statistic *p* value <0.05 or I^2^ > 75% [20]. For pleiotropy tests, we investigated horizontal pleiotropy using either the MR-Egger regression (intercept close to zero) or the MR pleiotropy residual sum and outlier (MR-PRESSO) technique (*p* value <0.05) [21]. We generated IVW radial variants and MR-Egger radial variants for improved visualization of IVW and MR-Egger estimates [22].

We also employed the Bayesian weighted Mendelian randomization (BWMR) method to further assess pleiotropy [23]. BWMR addresses the challenges of polygenicity and pleiotropy by incorporating Bayesian weighting and outlier detection. This method enhances the robustness of causal inference by accounting for the uncertainty of weak effects and detecting violations of the IV assumption due to pleiotropy. The BWMR analysis yielded consistent positive results, further supporting the causal link between RA and AF.

### 2.5. Power Calculations

We utilized the MR online calculator available at https://cnsgenomics.com/shiny/mRnd/ to estimate statistical power. For the binary outcome (AF), we considered the total number of cases, the proportion of phenotypic variance explained by genetic variants (*R*^2^) for RA, and a type one error rate of 0.05 in our calculations.

The power calculations indicated that our study had 81% power to detect an odds ratio (OR) ranging from 1.01 to 1.41 for the association between RA and AF. This high power suggests that our findings are robust and less likely to be due to random chance. The proportion of variation in the exposure variable (RA) explained by the genetic predictor (*R*^2^) is a critical factor in determining the power of the study. In our analysis, the selected SNPs collectively explained 4.9% of the variation in RA within the population.

The true causal association between the exposure (RA) and the outcome (AF) also influences the power of the study. A higher true causal effect size would result in greater power to detect the association. Our power calculations accounted for the sample size, the proportion of phenotypic variance explained by the genetic variants, and the expected effect size, ensuring that our study design was adequately powered to detect the causal relationship between RA and AF.

All analyses were performed using R software (Version 4.2.1) and relevant R packages. Statistical significance was defined as a two-sided *p* value <0.05.

## 3. Results

### 3.1. Instrumental Variables Validity

Five SNPs strongly associated with RA were identified as IVs in the MR analysis, following a rigorous selection process to ensure IV eligibility and exclude potential pleiotropic SNPs (Table 2). These five SNPs collectively explain 4.9% of the variation in RA within the population. The IV was robust, effectively ruling out the possibility of a null association due to instrument bias, as evidenced by the high F-statistics ranging from 1843 to 2701 for these SNPs [24]. According to power calculations, this study had 81% power to detect an odds ratio (OR) ranging from 1.01 to 1.41 for the association between RA and AF.

### 3.2. Effect of Genetically Predicated RA Variation on AF

**Figure 1:**
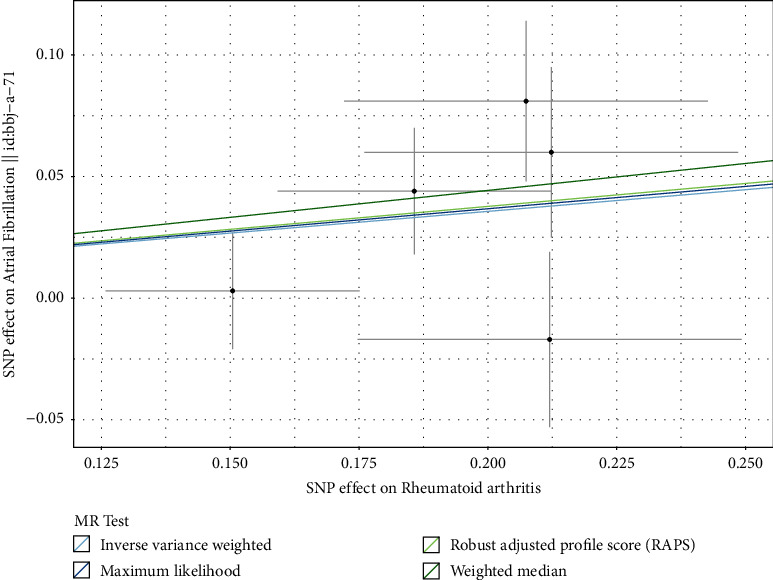
Scatter plot of SNPs associated with RA and the risk of AF. The plot related the effect sizes of the SNP-RA association (*x*-axis,) and the SNP-AF associations (*y*-axis) with 95% confidence intervals. The regression slopes of the lines correspond to causal estimates using four Mendelian randomization methods (the inverse variance weighted method, robust adjusted profile score (MR-RAPS), maximum likelihood, and the weighted median). Abbreviations: AF, atrial fibrillation; RA, rheumatoid arthritis; MR, Mendelian randomization; SNP, single-nucleotide polymorphism.

Principal causal relationship estimates indicate that RA increases the incidence of AF (IVW: OR = 1.20, 95% confidence interval [CI]: 1.01–1.41, *p* < 0.03). Consistent results were observed across three other methods, as summarized in Table 3 (MR-RAPS: OR = 1.21, 95% CI: 1.03–1.42, *p* < 0.02; maximum likelihood: OR = 1.20, 95% CI: 1.04–1.39, *p* < 0.01; WM: OR = 1.25, 95% CI: 1.03–1.52, *p* < 0.03; and BWMR: OR = 1.20, 95% CI: 1.02–1.42, *p* < 0.03), providing robust support for the causal relationship between RA and AF.  Figure 1 illustrates that the risk of AF escalates proportionally with the significance of IVs associated with RA.

### 3.3. Sensitivity and Heterogeneity Analysis

**Figure 2:**
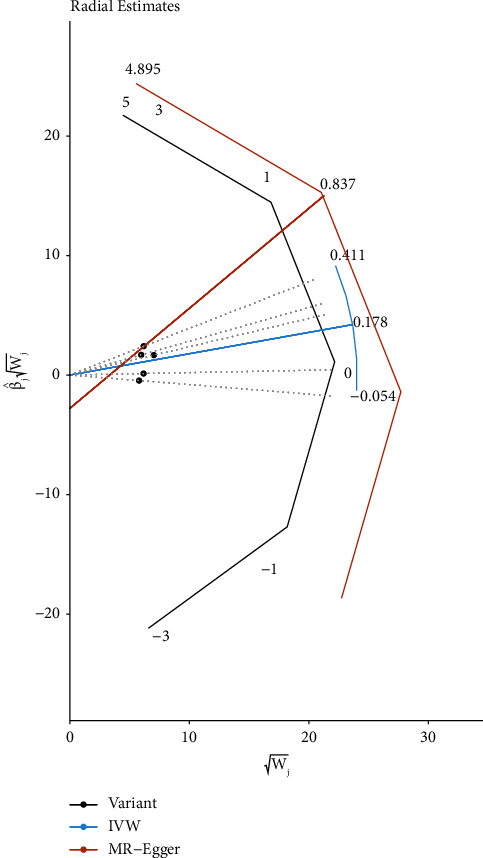
Radial plots to visualize individual outlier single nucleotide polymorphisms (SNPs) in the Mendelian randomization (MR) estimates for association between RA with AF. Black dots show valid SNPs and purple dots display invalid outlier SNPs. There is no significant outlier SNP in present plots. IVW indicates inverse variance weighted.

Cochran's *Q* statistics from the MR-Egger (*Q* = 4.857; *p*=0.183 > 0.05) and IVW (*Q* = 5.658; *p*=0.226 > 0.05) methods did not reveal significant heterogeneity. Both the MR-Egger test (intercept = −0.087, *p*=0.533) and MR-PRESSO test (global test *p*=0.307) indicated no directional pleiotropy. In the MR-PRESSO analysis and radial plot (Figure 2), no outlier SNPs were identified, indicating the absence of pleiotropic bias. In addition, leave-one-out analysis demonstrated that no individual SNPs significantly influenced the relationship between RA and AF (Supplement Figure 1).

## 4. Discussion

The causal relationship between RA and AF in the East Asian population remains largely unexplored. Our current MR study suggests that RA may indeed elevate the risk of AF in this population. Through multiple sensitivity analyses, we ensured that potential causal associations were not influenced by horizontal pleiotropy, thereby affirming the robustness of our effect estimates.

Three retrospective cohort studies involving 40,000 RA patients and more than 4 million non-RA controls assessed the potential link between RA and AF [6, 25, 26]. The meta-analysis of these studies showed that RA patients had a 30% higher risk of developing AF than non-RA participants [27]. In addition, recent studies have shown that RA patients have significant atrial remodeling. A correlation between the CRP level and prolonged electromechanical delays and impaired left atrial mechanical functions was found in RA patients [28]. RA is associated with a higher risk of developing AF, but causality for their associations is still unknown. In order to eliminate residual confounding, we made causal inferences for the associations between RA and the risk of AF using genetic variants linked to RA as the unconfounded proxy. In this MR study, we discovered a causal link between RA and AF.

Several biological mechanisms may underlie the effect of RA on the risk of AF. The systemic inflammation characteristic of RA, driven by the overexpression of inflammatory factors such as tumor necrosis factor (TNF) and interleukins (ILs), is believed to alter atrial electrophysiology and structural substrates, thereby increasing susceptibility to AF [29]. Independent associations have been observed between AF and SNPs of inflammatory-related genes such as interleukin-1 (IL1), IL-6, and IL-10 [30]. In addition, inflammation mediators and immune cells have been implicated in atrial electric remodeling, altering ion channels, calcium homeostasis, and conduction properties of the atria. Such structural remodeling leads to atrial dilation, fibrosis, apoptosis, and myolysis of cells, further predisposing to AF [31]. Moreover, TNF, IL-2, and platelet-derived growth factor have been shown to impact calcium homeostasis in cardiomyocytes, shortening action potential duration, and increasing the initiation of arrhythmias [32].

Recent studies have highlighted the role of chronic inflammation in promoting atrial structural remodeling. Inflammatory cytokines such as TNF and IL-6 can induce fibroblast proliferation and extracellular matrix deposition, leading to atrial fibrosis [33]. This fibrotic remodeling disrupts the normal atrial architecture, creating a substrate for reentrant circuits and increasing the likelihood of AF [34]. In addition, inflammation-induced oxidative stress can damage atrial myocytes, further contributing to structural remodeling and AF susceptibility [35].

Inflammation also affects the autonomic nervous system, which plays a crucial role in the initiation and maintenance of AF. Elevated levels of inflammatory cytokines have been associated with increased sympathetic activity and reduced vagal tone, creating an imbalance that favors AF development. Moreover, studies have shown that RA patients exhibit altered expression of ion channels, such as reduced L-type calcium channel expression and increased expression of proarrhythmic potassium channels, which can promote atrial arrhythmogenesis [36].

Overall, the interplay among systemic inflammation, structural remodeling, and autonomic dysfunction creates a proarrhythmic environment in RA patients, increasing their susceptibility to AF. Understanding these mechanisms is essential for developing targeted therapies to mitigate AF risk in this population [36].

Our study possesses several strengths. First, it is the first MR study in the East Asian population to systematically investigate the causal relationship between RA and the risk of developing AF. Second, MR, by assigning alleles of SNP sites randomly much earlier than the occurrence of potential confounding factors, minimizes the influence of confounding factors and reverse causality. Lastly, we employed various MR methods to collectively assess the robustness of our results.

However, there are notable limitations to our study. First, as our MR analysis was conducted specifically on an East Asian population, generalization to non-East Asian populations should be done cautiously. Second, the GWAS summary statistics for AF lacked information on AF subtypes, precluding an examination of the relationship between RA and specific AF subtypes. Future research should aim to assess the association between RA and different AF subtypes. Third, while we attempted to mitigate genetic pleiotropy by excluding known SNPs associated with AF and confounder factors prior to our MR analysis, we cannot entirely rule out the possibility of genetic pleiotropy.

There are potential differences in genetic backgrounds and environmental factors between East Asian and non-East Asian populations that may influence the association between RA and AF. Genetic variations and environmental exposures can differ significantly across populations, potentially affecting the generalizability of our findings. For instance, lifestyle factors, dietary habits, and healthcare access can vary widely between populations, influencing the prevalence and manifestation of both RA and AF. In addition, genetic diversity and population-specific genetic variants may contribute to differences in disease susceptibility and progression. Therefore, it is crucial to validate our findings in diverse ethnic groups to ensure their applicability across different populations.

## 5. Conclusions

In conclusion, our findings provide compelling evidence for a causal link between RA and AF in the East Asian population, underscoring the importance of addressing inflammation in the management of AF among RA patients. Further research is warranted to validate these findings in diverse populations and to elucidate the precise mechanistic pathways underlying this association.

## Figures and Tables

**Table 1:** Data sources of summary datasets.

Contribution	Traits	Sample size	nSNPs	Population
Exposure	Rheumatoid arthritis	212,453	8,885,805	East Asian
Outcome	Atrial fibrillation	36,792	5,018,048	East Asian

**Table 2:** Characteristics of SNPs associated with rheumatoid arthritis.

SNP	EA	OA	Beta	EAF	SE	*P* value	*R*-squared	*F*-statistic
rs12612769	C	A	0.1505	0.3029	0.0247	1.06*E* − 09	0.0096	2051
rs3757387	C	T	0.2123	0.1068	0.0363	4.83*E* − 09	0.0086	1843
rs56139217	C	T	0.2120	0.1138	0.0373	1.26*E* − 08	0.0091	1944
rs79658451	C	G	0.2074	0.1158	0.0353	4.19*E* − 09	0.0088	1888
rs80202727	T	C	0.1857	0.2392	0.0265	2.47*E* − 12	0.0126	2701

SNP indicates single nucleotide polymorphism; EA, effect allele; OA, other allele; EAF, effect allele frequency; SE, standard error.

**Table 3:** MR estimates from 4 methods of assessing the causal effect of rheumatoid arthritis on the risk of atrial fibrillation.

Method	nSNPs	Beta	SE	*P* value	OR (95% CI)
Inverse variance weighted	5	0.18	0.08	0.03	1.20 (1.01–1.41)
Robust adjusted profile score (RAPS)	5	0.19	0.08	0.02	1.21 (1.03–1.42)
Maximum likelihood	5	0.18	0.07	0.01	1.2 (1.04–1.39)
Weighted median	5	0.22	0.10	0.03	1.25 (1.03–1.52)
BWMR	5	0.19	0.08	0.03	1.20 (1.02–1.42)

SNP, single-nucleotide polymorphism; SE, standard error; OR, odds ratio; CI, confidence interval; BWMR, Bayesian weighted Mendelian randomization.

## Data Availability

Publicly available datasets were analyzed in this study. These data can be found at https://gwas.mrcieu.ac.uk/.

## Supplementary Materials

Supplementary Materials Supplement Figure 1: leave-one-out analysis of Mendelian randomization (MR) estimates of genetic risk of rheumatoid arthritis on atrial fibrillation.
