# Supplementary material for: Investigating the Causal Link between Rheumatoid Arthritis and Atrial Fibrillation in East Asian Populations: A Mendelian Randomization Approach
Source: Cardiol Res Pract. 2024 Jul 15;2024:3274074. doi: 10.1155/2024/3274074 (PMC11262875; doi:10.1155/2024/3274074)
Supplement: Supplementary Materials — Supplement Figure 1: leave-one-out analysis of Mendelian randomization (MR) estimates of genetic risk of rheumatoid arthritis on atrial fibrillation. [file 3274074.f1.docx]

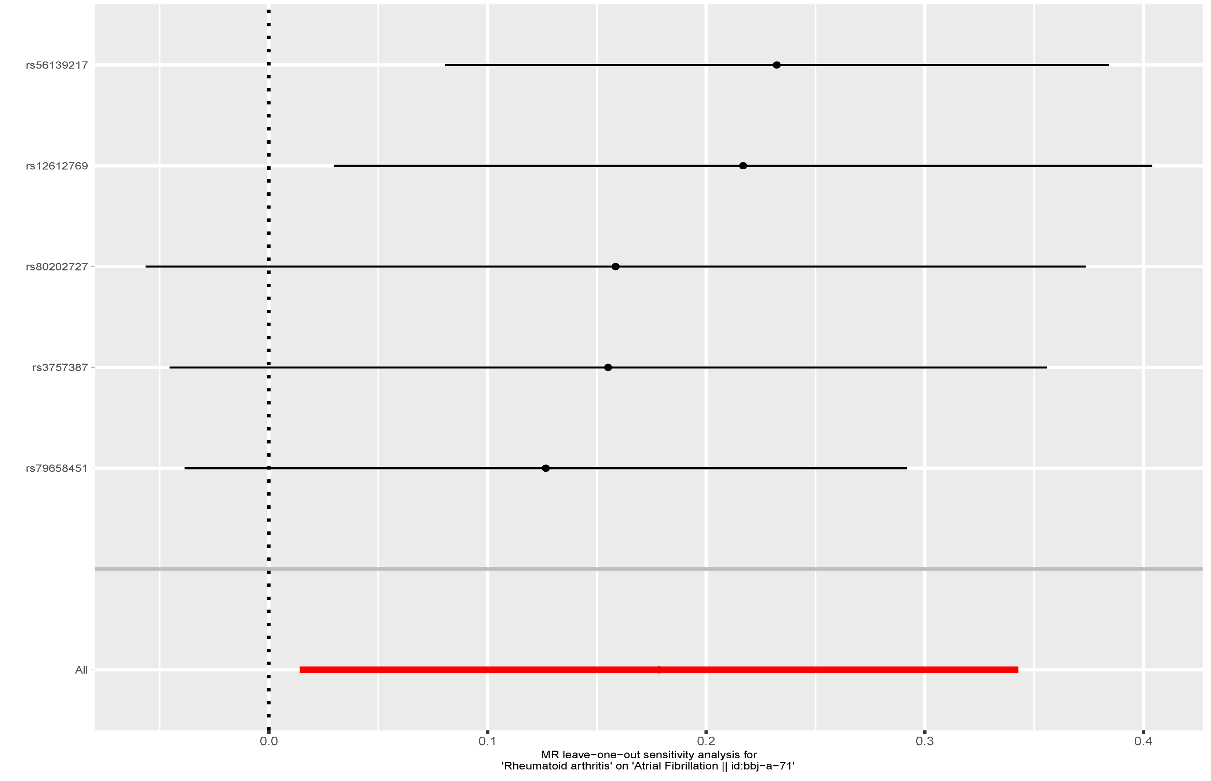


Supplement Figure 1: Leave-one-out analysis of Mendelian randomization (MR) estimates of genetic risk of rheumatoid arthritis on atrial fibrillation. Black boxes corresponding to each of the single nucleotide polymorphisms (SNPs) denote odds ratios (OR) derived from inverse variance weighted (IVW) after leaving the corresponding SNP in turns. The red box corresponding to ‘ALL’ indicates the pooled IVW MR estimate. Horizontal lines denote a 95% confidence interval (CI).
